# Recent Trends and Opportunities for the Targeted Immuno-Nanomaterials for Cancer Theranostics Applications

**DOI:** 10.3390/mi13122217

**Published:** 2022-12-14

**Authors:** Clyde John, Kaahini Jain, Hema Brindha Masanam, Ashwin Kumar Narasimhan, Arutselvan Natarajan

**Affiliations:** 1 Department of Molecular and Cellular Biology, University of Illinois Urbana-Champaign, Urbana, IL 61801, USA; 2 Department of Neuroscience, Boston University, Boston, MA 02215, USA; 3 Advanced Nano-Theranostics (ANTs), Biomaterials Lab, Department of Biomedical Engineering, SRM Institute of Science and Technology, Kattankulathur, Chennai 603203, Tamil Nadu, India; 4 Molecular Imaging Program at Stanford (MIPS), Department of Radiology and Bio-X Program, Stanford University, Stanford, CA 94305, USA

**Keywords:** immunotherapy, immune checkpoint inhibitors, immuno-nanomaterials, immunodiagnostics

## Abstract

The targeted delivery of cancer immunotherapies has increased noticeably in recent years. Recent advancements in immunotherapy, particularly in blocking the immune checkpoints (ICs) axis, have shown favorable treatment outcomes for multiple types of cancer including melanoma and non-small-cell lung cancer (NSLC). Engineered micromachines, including microparticles, and nanoplatforms (organic and inorganic), functionalized with immune agonists can effectively deliver immune-targeting molecules to solid tumors. This review focuses on the nanomaterial-based strategies that have shown promise in identifying and targeting various immunological markers in the tumor microenvironment (TME) for cancer diagnosis and therapy. Nanomaterials-based cancer immunotherapy has improved treatment outcomes by triggering an immune response in the TME. Evaluating the expression levels of ICs in the TME also could potentially aid in diagnosing patients who would respond to IC blockade therapy. Detecting immunological checkpoints in the TME using noninvasive imaging systems via tailored nanosensors improves the identification of patient outcomes in immuno-oncology (IO). To enhance patient-specific analysis, lab-on-chip (LOC) technology is a rapid, cost-effective, and accurate way of recapitulating the TME. Such novel nanomaterial-based technologies have been of great interest for testing immunotherapies and assessing biomarkers. Finally, we provide a perspective on the developments in artificial intelligence tools to facilitate ICs-based nano theranostics toward cancer immunotherapy.

## 1. Introduction

The immune system defends the body against pathogens, which suggests extensive implications for its use in therapeutic interventions. For one, many characteristics of cancer cells are similar to those of healthy cells, thus making it difficult for the immune system to identify them as foreign elements. It also enables the biomolecules expressed by tumors at the tumor microenvironment (TME) to evade detection by immune cells. Cytokines may even form a tumor-supportive immune microenvironment that inhibits antitumor activity and transmits signals that directly promote tumor growth [1,2,3]. Therefore, new treatment strategies are required to overcome these challenges in detecting cancer cells and thereby preventing tumor development.

Cancer immunotherapy is a treatment method in which the immune system is stimulated to communicate with the tumor at the TME to detect, target, and destroy the growth of the cancer cells or malignancy [4]. Currently, several clinical studies are using immunotherapy as a first line of treatment for cancer, which has enormous potential in comparison to conventional treatments such as surgery, chemotherapy, and radiotherapy [5]. Additionally, immunotherapies are showing significant advancements in the treatment of solid tumors. 

A recent report on immune drugs, in which monoclonal antibodies (mAbs) were used for solid tumors in poor-prognosis patients, showed a 20% increased survival when compared to chemo- and radiotherapy. Patients lived longer when monoclonal antibodies, molecular inhibitors, or chemotherapy were given directly to the tumour site [6]. However, immunotherapy is not widely accessible to patients due to the high cost—up to USD 250,000 per patient [7]. Consequently, tailored immune drug administration is an essential treatment method for solid cancer therapy and should be integrated into ongoing clinical trials.

In this review, we have outlined multiple diagnostic imaging platforms with targeted nanomaterials that allow for the selection of patients shows positive results from the use of noninvasive immunotherapy. In this treatment, target-specific activation of immunogenic antigens initiates a defensive effector immune response that kills specific cancer cells. Various diagnostic imaging systems can be used to measure immune activation in the TME, which provides a better prognosis for cancer [8].

### 1.1. Challenges in Immunotherapy

Cancer immunotherapy based on immune checkpoint inhibition (ICI) for patient screening remains complex in terms of clinical decision-making. ICI therapy is only effective for patients with mismatch repair deficit (dMMR) or high-microsatellite instability (MSI-H) in their metastatic colorectal cancer (mCRC) [9]. In addition, neurological immune-related adverse events (irAEs) are increasingly common in oncologic treatment approaches, especially in patients treated with ICIs and combination therapy [10]. The immune-related response criteria (irRC) clearly define an important concept necessary for the assessment of immune-related responses; however, several pitfalls and issues related to irRC remain to be solved [11]. 

Cancer treatments using ICI have short-term and long-term side effects, and many long-term, or chronic, side effects are currently unfolding. A new study reported that ICI can cause a range of long-term side effects, but most of them are mild, with more than 40% of the patients affected [12]. These chronic effects were skin rashes, hypothyroidism, and joint pain. Monitoring immune checkpoint blockade (ICB) therapy can provide more information to address these issues. Measuring IC expressions in the TME over time could provide clinicians with useful information for evaluating potential outcomes. In addition, biomarkers that can predict therapy response to ICB can be utilized for further advanced-precision immunotherapy. 

### 1.2. Need for Targeted Cancer Immunotherapy

Treatment response by immunotherapeutic drugs is characterized by immune cell activation, which can have adverse effects on normal cells. Hence, a controlled mechanistic pathway is required to understand the genomic sequence and mutations in the tumor to prescribe treatments. Effective transport of cancer-immune drugs at the molecular level can be delivered through engineering nano- and microcarriers functionalized using targeted moieties. However, the regulated release kinetics of immune drugs at the intra-tumoral region would follow burst release than controlled fashion. To improve drug release patterns, externally triggered sources across electromagnetic regions, such as photothermal, sound, and radiation (X-rays and radiofrequency), are utilized for personalized medicine.

**Figure 1:**
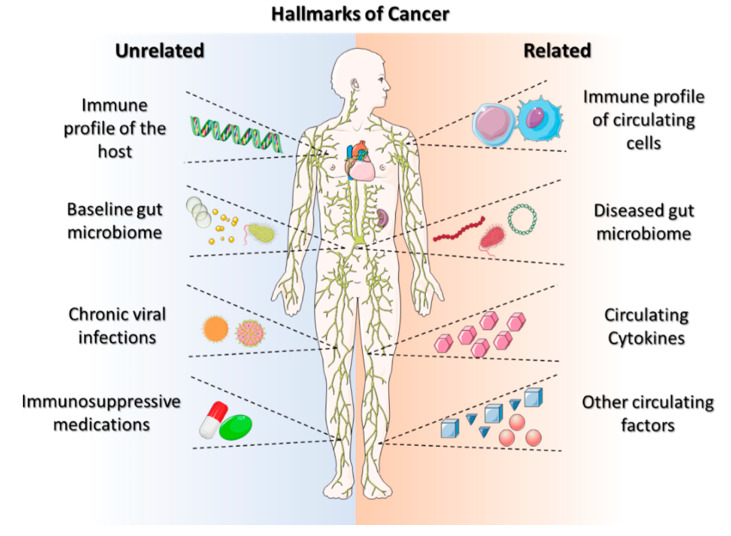
Schematic representation of cancer hallmarks for the immunological component of a tumor. Modified from Ref. [13].

The ability of solid tumors to bypass the body’s antitumor immune response is becoming a hallmark characteristic of cancers (Figure 1). Galluzzi et al. defined the hallmarks of anticancer immunotherapy as immunogenicity, immunosuppression, susceptibility, composition, localization, and functionality [13]. These hallmarks of anticancer immunotherapy can lead to a successful treatment modality for personalized care. Systemic activation reduces the sensitivity of immunotherapies and potentially hinders the anticancer-immune-drug response [14]. The beneficial effects of anticancer immunotherapy are dependent on (i) their innate capacity to elicit a tumor-targeting immune response, (ii) their capacity to establish an immunosuppressive TME, and (iii) their sensitivity to immune-effector mechanisms [15]. These hallmarks aid in developing personalized immunotherapeutic regimes, requiring improved effectiveness and multiparametric evaluations. However, there is a need to search for the hallmark identifications that improve immune-based therapeutic outcomes, beginning with the identification and usage of ICIs, which provide prognostic insights into cancer immunotherapy.

### 1.3. Nanotechnology in Cancer Immunotherapy

Nanomaterials ranging from 1 to 100 nm were used for immunotherapy with combinational drugs for effective cancer treatment [16]. Typically, these targeted nanoparticles (NPs) were composed of a drug-concentrated core and a functionalized outside layer, or shell. These engineered nanoparticles were further optimized based on size, shape, and surface properties to increase their efficacy and reduce side effects. Nanomaterials with different compositions were used to generate an effective cancer immunotherapy based on ICIs. 

Anticancer drugs currently used in clinical settings are hydrophobic, thereby making it hard for them to travel in aqueous-body environments [17]. Encapsulation is one strategy used for loading hydrophobic drugs in a nanoparticle carrier. This increases the concentration of hydrophobic drugs in the human system by a factor of over 5 × 10^4^ [18]. Hydrophilic drugs come in the form of both macromolecules and small molecules, with some limitations. For instance, cellular uptake is poor because they struggle to cross hydrophobic membranes, have low bioavailability, and have a short half-life. However, this has been combated through the use of nanoparticles, which protect and deliver hydrophilic drugs. 

“Naturally targeted” immune cells, such as macrophages, monocytes, neutrophils, and dendritic cells (DCs), phagocytose the free NP drugs to transport them to the cancer site. Targeting cancerous cells using selective binding to specific receptors is possible due to ligands such as small organic molecules, peptides, antibodies, and nucleic acids. This design allows for the transport and simultaneous delivery of multiple drugs to a target, which is useful for multimodal therapy techniques. Through the enhanced permeability and retention (EPR) effect, NPs can easily drive into the TME. NPs are a flexible platform to use in cancer immunotherapy, displaying potential in a variety of fields such as imaging and drug delivery without harming another internal system [19]. Thus cancer immunotherapy is a cutting-edge treatment that dynamically changes the immune system’s ability to fight cancer [20].

## 2. Immune-Targeting Nanomaterials

**Figure 2:**
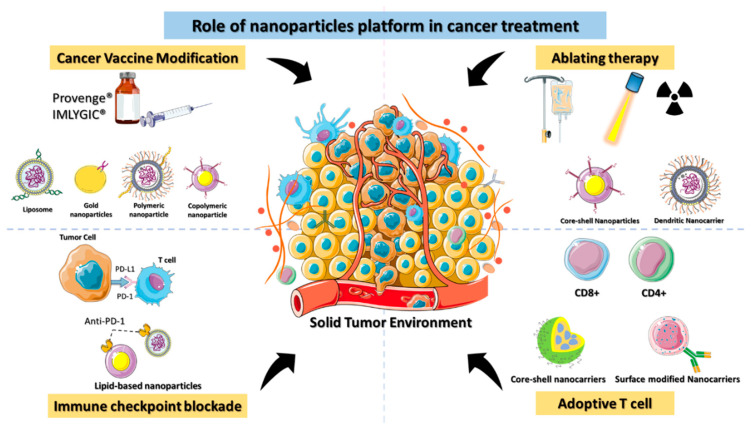
Schematic depiction of the role of nanomaterials platforms in various immunotherapies for cancer.

Immunotherapy can be classified as either active or passive immunotherapy. Active immunotherapy directly stimulates the immune system to target cancer cells, whereas passive immunotherapy activates immune cells through external agents like monoclonal antibodies, drugs, and cytokines. Cancer immunotherapy remains challenging, however, as it can have life-threatening side effects due to the severity of the disease [21]. The lack of targeting is the fundamental cause of immunotherapy failure against cancer. Nontargeted ICIs elicit a critical immunological response in the host that affects even normal cells. Immunotherapy must be delivered in a precise way to get the optimal therapeutic effect in cancer patients [22]. Clinical trials of cancer immunotherapies have shown promising outcomes for immunological checkpoint inhibition, adoptive T cell therapy, therapeutic cancer vaccines, and ablation-enhanced immunogenic cell death (Figure 2). Coloading of drugs into a nanocarrier platform can efficiently deliver the drug to the subcellular levels of cancer residing in the TME [23].

### 2.1. Nanocarriers for Immune Checkpoint Inhibitor (ICI) or Blockade (ICB)

Recently, cancer immunotherapies using ICIs or ICBs have shown remarkable advancements in the treatment of advanced-stage cancers, displayed by improved patient outcomes. When these ICIs bind to the surface of partner proteins such as T cells, they turn “off” T cell signals and render them inactive. In other words, the ICIs inhibit cytokine expression, which prohibits a proper immune response from the T cell. ICIs block the checkpoint proteins of the tumor cells from binding to the ligand, resulting in a deeply improved prognosis. The ability of ICIs to regulate both innate and adaptive immune cells allows them to effectively renew the immune response and restore tumor-cell infiltration [24]. Research shows that sixteen FDA-approved immunomodulators, nine ICIs, and multiple cytokines and adjuvants are available as cancer immunotherapies [25].

Throughout the decade, many mAb-based ICI drugs have shown remarkable results towards several IC targets, e.g., cytotoxic T lymphocyte-associated protein 4 (CTLA-4), and programmed cell death 1 or ligand 1 and 2 (PD-1 or PD-L1 or PD-L2) show successful results in the clinic [26]. These induce remarkable tumor regression and long-lasting recovery in certain cancer patients. For example, acute lymphoblastic leukemia (ALL) is highly cancer-resistant, but T cells expressing chimeric antigen receptors (CAR) have shown improved prognosis [27]. In clinical trials, a variety of immunostimulatory mAbs, small molecules that reverse cancer-associated immunosuppression, and tumor-targeting therapeutic vaccines are used for treatments. Several immunotherapeutic cancer treatments are becoming more widely used and some ICIs have even reached late-stage clinical testing.

**Figure 3:**
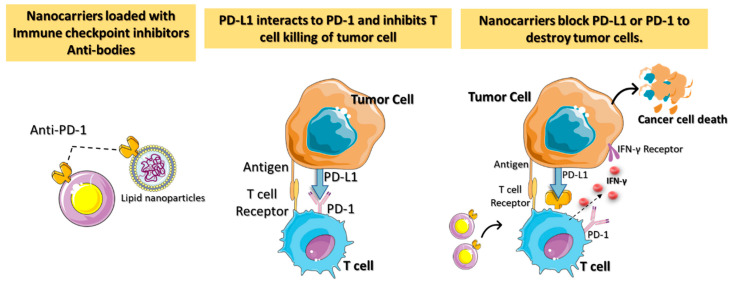
A diagrammatic representation of the immune checkpoint’s blockage using nanocarriers loaded with checkpoint inhibitors and antibodies for targeted cancer immunotherapy. Antitumor immune responses are regulated by proteins called checkpoints, such as PD-L1 on tumor cells and PD-1 on T cells. When PD-L1 binds to PD-1, it inhibits the activity of T cells and prevents them from killing tumor cells. Targeted nanocarriers functionalized with an immune checkpoint inhibitor (anti-PD-L1 or anti-PD-1) allow T cells to destroy tumor cells by preventing PD-L1 from attaching to PD-1.

When utilizing nontargeted drug delivery, patients may be at risk for developing autoimmune reactions and related adverse effects [28]. Critical challenges in ICI-based cancer immunotherapies include targeted localization, protein infusion stability, and regulated ICI release. ICI blockage of the receptor on T cells results in more peripheral T cell recruitment, leading to an autoimmune reaction [29]. Nanotechnology-assisted administration of ICIs has benefits, including enhanced drug accumulation in tumors, simultaneous delivery of multiple ICIs, real-time delivery monitoring, and even facilitating advanced delivery techniques. The engineered nanoparticles with ICIs are better able to reach the tumor via enhanced permeability and retention (EPR) effects. The nanomedicine-based ICIs can penetrate complicated tumor environments by overcoming physiological barriers such as the blood–brain barrier and the host immune response (Figure 3).

Polo-like kinase 1 (PLK1) enzymes are key mitotic kinases that are overexpressed in a variety of cancers and exhibit oncogenic properties. PLK1 inhibition kills cancer cells and upregulates PD-L1 expression in the TME through the mitogen-activated protein kinase (MAPK) pathway. Yantesee et al. developed a nanoplatform strategy to express the relationship between PD-L1 antibody and PLK-1 inhibitor to target ICIs to improve the survival of NSCLC. The nanoplatform consists of mesoporous silica nanoparticles (MSNs) loaded with the immunotherapy drug Volasertib (PLK-1 inhibitor) and functionalized with PD-L1 on the surface of nanostructures to target cancer cells. This combination of strategies is defined as the ARAC platform (antigen-release agents and a checkpoint). The increase in the uptake of MSNs in NSCLC via PD-L1, thereby delivering PLK-1 in the cytosol, leads to cellular apoptosis. In vivo evaluation of ARAC has shown an improved survival rate (up to 30 days) and a threefold reduction in the tumor volume compared to Volasertib. Inhibiting PLK1 results in an increase in PD-L1 expression in the remaining surviving cancer cells. The PD-L1 antibody-targeted ARAC significantly enhances adaptive antitumor immunity and T cell activity by elevating the CD8^+^ ratio and tumor infiltration. The subsequent dosage of ARAC results in immunosuppression in the TME [30].

A decline In the efficacy of ICBs in cancer immunotherapy is often attributed to tumor suppressors, which act as the driving force behind tumorigenesis. Despite the cell-autonomous tumor-suppressive effects, evidence indicates that the p53 protein modulates the TME by altering the contact of tumor cells with immune cells. When p53 is restored genetically, myeloid cell activation is upregulated, and tumor antigen-specific adaptive immunity is enhanced. Shi et al. developed specific hybrid nanoparticles with a lipid-based poly (lactic-co-glycolic acid) (PLGA) polymer core to transport p53 mRNA transfection complexes [31]. Targeted p53 mRNA nanoparticles suppressed the pro-tumorigenic M2-type tumor-associated macrophage (TAM) and enhanced the MHC-1 expression with antitumor immunity. Glycogen synthase kinase 3 (GSK-3) works as an unanticipated tumor suppressor in certain cancers [32], as it is responsible for phosphorylating the oncoprotein known as mouse double minute 2 homologs (MDM2). The MDM2 protein is the most important regulator of the p53 protein [33]. Thus, instead of restoring the p53, direct inhibition of GSK3 effectively reduces PD-1 expression and promotes the proliferation of cytotoxic T cells, resulting in long-term T cell memory. However, delivery of the GSK-3 small molecule is challenging due to its relatively short half-life and high availability in off-targeted organs. Thierry et al. reported a nano formulation containing a GSK-3 inhibitor loaded with PEG-PLGA nanoparticles to block the PD-L1 ICP [34]. Results indicate that the nanoplatform enhances the efficacy of the ICB therapy, thereby improving cancer treatment.

### 2.2. Nanocarrier for Adoptive Cell Transfer (ACT)

Adoptive cell therapy (ACT) involving T cells derived from tumor-infiltrating lymphocytes (TIL) has the potential for both immunostimulatory and immunosuppressive cancer therapies [35]. However, ACT is still a barrier to a robust activation of the host immune system, the regulation of signaling pathways, and the maintenance of a progression-free state. The use of the nanoplatform can improve the production, application, and efficacy of T cell immunotherapy for a variety of cancer conditions [36].

**Figure 4:**
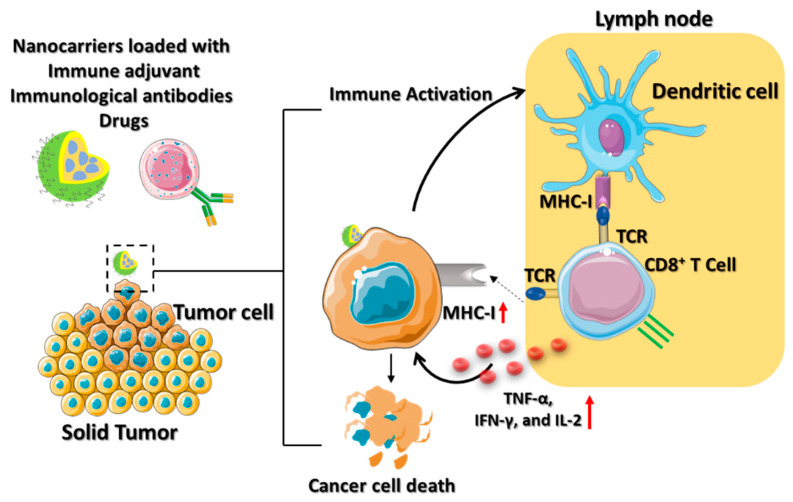
Schematic illustration which depicts the mechanism of ACT using various nanocarriers. The nanocarriers, such as lipid-based nanoparticles, nanodiscs, or core–shell nanoparticles are coloaded with immune drugs, antibodies, and adjuvants for active cancer immunotherapy. The immune activation of the dendritic cells contributes to immunological responses to the TME by secreting IFN-γ, which is promoted by the effector activities of CD8^+^ T cells to cause tumor death.

Cisplatin, also known as cis-diamminedichloroplatinum (II) (CDDP), is an effective cancer drug in clinical trials [37]. The effectiveness of cisplatin in inhibiting tumor growth has been established; however, its organ toxicity presents a significant challenge. Yao and his colleague utilized the CDDP drug-loaded polyethylene glycol (PEG)–polyglutamate block copolymer micelle nanoparticles (CDDP-NPs) for antitumor immune response [38]. The longer retention time of the CDDP-NPs in the TME upregulates the major histocompatibility complex class I (MHC-I), leading to the activation of the amyloid precursor protein (APP) pathway. The DCs are attributed to the interaction between the T cell receptor (TCR) and the peptide it recognizes by the APP (Figure 4). Thus, the CDDP-NPs indirectly activate the tumor antigen-presenting CD8^+^ T cell through the TCR signaling pathways. The OX40 is a tumor necrosis factor receptor superfamily (TNFRSF) domain that acts as a costimulatory antigen molecule expressed in the CD8^+^ T cells [39]. Therefore, the use of CDDP-NPs along with the OX40 agonists (aOX40) had a remarkable therapeutic effect in inducing T cell infiltration in the tumor environment. The promotion of aOX40 would activate the proinflammatory cytokines and secrete TNF-α, IFN-γ, and IL-2 for killing the tumor. Immunotherapies utilizing CDDP-NPs remain in the preclinical phase of development. It is crucial to analyze the efficacy of nanomedicine in the clinic to determine the possible role of T cell infiltration in the tumor.

The development of resistance to tumors can also be through the stimulation of the innate immune system. The development of active nanocarriers facilitates binding with the innate immune system to regulate “danger signals”. The activation of danger signals at the tumor site by Toll-like receptors such as retinoic-acid-inducible gene I (RIG-I), nucleotide-binding oligomerization domain (NOD), and stimulator of interferon genes (STING) improves T cell response [40]. Current clinical research indicates that natural ligands for STING and cyclic dinucleotides (CDNs) are effective agonists for inducing potent antitumor immunity. CDNs are susceptible to nucleases and impermeable to cell membranes, which hinders their systemic delivery. Irvine et al. formulated PEGylated lipids conjugated with CDNs that self-assemble in lipid nanodiscs (LNDs) [41]. Discoid-shaped nanoparticles (LNDs-CDNs) are optimal for intravenous delivery and show effective penetration within the tumor model that leads to tumor necrosis.

Effective cancer cytosol penetration of CDN-loaded lipid nanomaterials triggers antitumor immunity through CD8^+^ T cell and natural killer (NK) cell activation [42]. STING activation pathways trigger NK cells to produce interferon γ (IFN-γ), promoting PD-1 expression in tumor cells [43]. However, the data from studies imply that a single dose is insufficient to activate NK cells [44]. Therefore, a combination of PD-1 antibody with the CDN agonists loaded in lipid nanoparticles has an antitumor impact in lung metastasis under multiple-dose cycles. Ferroptosis is an iron-dependent form of apoptosis for tumor suppression. Cancer cells undergoing ferroptosis also trigger IFN-γ, which promotes the CD8^+^ T lymphocytes in the TME. The expression profile analysis identified miR-21-3p as the highly upregulated microRNAs that regulate ferroptosis. Chunying Li’s group examined the anti-PD-1 immunotherapy impact of miR-21-3p-loaded gold nanoparticles in a preclinical tumor model [45]. T-cell-adoptive cancer immunotherapies can be improved by integrating the nanosystem loaded with the immune drugs.

### 2.3. Nanocarriers for Cancer Vaccine

Cancer vaccines generally use tumor-associated antigens (TAAs) and tumor-specific antigens (TSAs) to boost the patient’s immune system. Therapies involving TSAs or neoantigens selectively expressed in the tumor have been at the forefront of cancer immunotherapy. Vaccines based on neoantigens have the potential to circumvent central immune tolerance and activate tumor-specific T cells [46]. Sipuleucel-T (Provenge^®^) and talimogene laherparepvec (IMLYGIC^®^) are some of the DCs-based cancer vaccines currently in phase I clinical trials [47,48]. However, poor targeting of the neoantigen-based cancer vaccines results in a low affinity to the TCR, making it incapable of mediating an effective antitumor response. Combining the NPs platform with the neoantigen-targeted vaccine delivery has therapeutic potential in cancer immunotherapy.

Jadidi and colleagues were the first to report that the combined suppression of lymphocyte-associated gene 3 (LAG3) and PD-1 by DC vaccines improves anticancer efficacy in the TME. LAG3 is an inhibitory receptor generated by immune cells to trigger T cell dysfunction [49]. Initiating immunosuppressive responses and promoting the proliferation of cancer cells, LAG3 promotes the development of cancer [50]. Specific siRNAs and LAG3/PD-1 antibodies were loaded onto chitosan–dextran sulfate–lactate (TMC-DS-L) nanoparticles, which led to increased IFN-γ production at the tumor site [51].

The primary obstacles in cancer immunotherapy are multiple drug resistance (MDR) and the inadequate accumulation of cancer vaccines in the TME [52]. Yao et al. developed a zwitterionic polymer employing poly (carboxy betaine methacrylate) (PBCMA) as a polymer shell over mesoporous organo silica nanoparticles (MONs) to overcome MDR in the TME [53]. PCBMA is loaded with redox-responsive sulfur dioxide (SO_2_), prodrug molecules (DN 2,4 dinitrobenzene-sulfonyl chloride), and chemotherapeutic drugs (DOX, doxorubicin) to treat MDR cancer effectively. The downregulation of P-glycoprotein in the presence of SO_2_ makes cancer cells more susceptible to chemotherapy-induced apoptosis. PCBMA increases the nanomedicine’s intratumor accumulation, resulting in a 94.8% reduction in tumor growth. The redox-responsive SO_2_ sensitizes cells, which aids DOX in prolonging the apoptosis in the TME.

**Figure 5:**
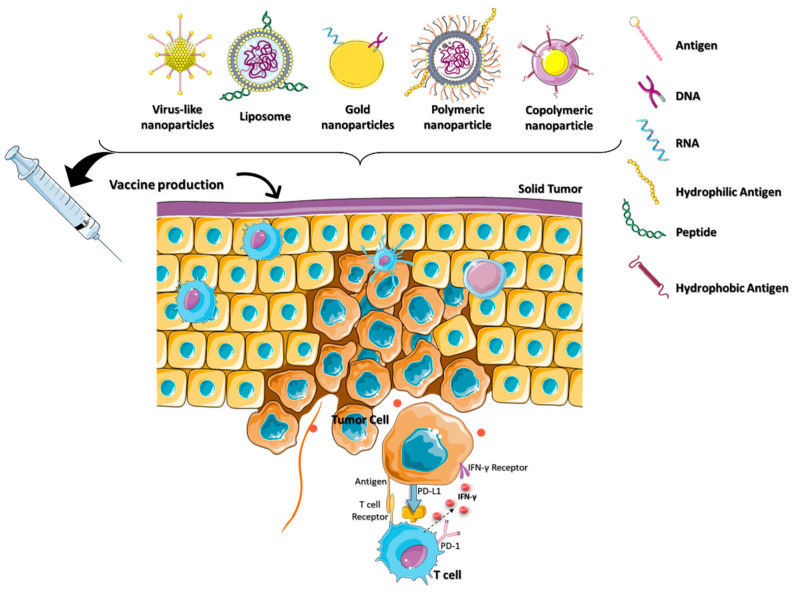
The cancer vaccination nanoparticles were camouflaged for specific functions in the TME. Nanovesicles containing a cancer vaccine comprised of a variety of antigens and biomolecules are being developed for use in cancer immunotherapy.

Preclinical immuno compatibility development requires the controlled activation of innate immunity [54]. Biopolymers (proteins, nucleic acids, collagen, chitosan, etc.), synthetic polymers (polystyrene, poly (lactic-co-glycolic acid) (PLGA), and poly (amino ester) (PBAEs)), and stimuli-responsive polymers loaded with immunoadjuvants have MDR capabilities that promote immunological activity. These polymers enable multi adjuvant synergy in cancer immunotherapy by stabilizing drug–cargo transport and activating proinflammatory cytokines. Thus, the camouflage of polymeric nanoparticles for the delivery of cancer vaccines provides an efficient platform for cancer immunotherapy (Figure 5).

### 2.4. Nanocarriers for Immunogenic Cell Death (ICD)

Immunotherapy is a treatment method to eradicate cancer cells with a high degree of specificity and minimal side effects while preventing their recurrence. Chemotherapy, photothermal therapy (PTT), radiotherapy, and reactive-oxygen-species (ROS)-mediated therapies can induce immunogenic cell death (ICD), which is used to improve cytotoxic-based antitumor immunity. Cancer immunotherapies have had limited success in ablation treatment due to the development of local and systemic immunosuppression and immune evasion. Local radiation therapy in the TME destroys cancer cells by activating the immune system [55]. When radiation and immunotherapy are combined on a nanoplatform, they have the potential to enhance the overall therapeutic efficacy [56]. These methods have the potential to improve patient outcomes, but they encounter challenges in optimizing the radiation dose, toxicity, and timing of combined therapies [57].

Chemotherapeutics can induce ICD and release tumor-specific antigens that are effective against numerous types of cancer [58]. Combining chemotherapeutics and immune adjuvants is a viable method for achieving synergistic therapeutic benefits. Deng et al. reported a nanocarrier composed of liposomal spherical nucleic acids (SNAs) and FDA-approved 1,2-dioleoyl-sn-glycerol-3-phosphoethanolamine (DOPE) for enhanced cellular absorption and stability against nucleases [59]. Conjugating chemotherapeutics such as doxorubicin (DOX) with DOPE triggered the ICD (DOPE-DOX) to secrete tumor-specific antigens. CpG oligodeoxynucleotides (CpGs) and matrix metalloproteinases-9 (MMP-9) are immunostimulatory reagents (DOPE-MMP-CpG) that amplify the immune response and promote immune cell penetration into the TME, respectively. Tumor cell MMP-9 and glutathione stimulated lipid-encapsulated SNAs to release DOX and CpGs into the TME. However, immunosuppression and cytotoxic side effects continue to restrict the use of these nanocarriers.

Yibru et al. developed a pH-responsive polyamidoamine (PAMAM) dendritic nanoparticles into the copolymer hydrogel (PCLA-PEG-PCLA) for the codelivery of DOX (PAMAM-DOX). PCLA-PEG-PCLA (poly(caprolactone-co-lactide)-poly (ethylene glycol)-b-poly(caprolactone-co-lactide) is a thermo reversible polymer ability to integrate with PAMAM-DOX nanocarriers for targeted drug delivery. At body temperature, PAMAM-DOX dendritic nanocarriers form a gel and facilitate a sustained DOX release in the TME. The combination of the immune activation with various cancer ablation techniques enhances the death of tumors. IND coupled with PAMAM-DOX also promotes the activate NK cells to attack tumor cells. However, immunological adjuvants in combination with chemotherapy remain resistant in treating some forms of cancer, especially triple-negative breast cancer (TNBC).

**Figure 6:**
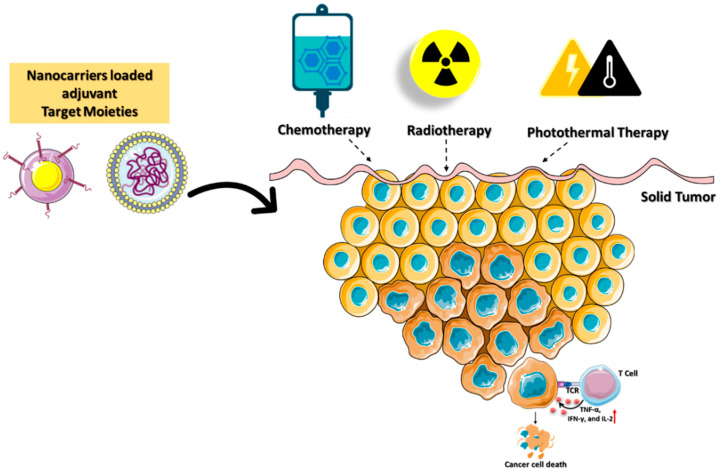
Schematic illustration of immunogenic cell death mediated by targeted nanocarriers conjugated with stimulating molecules such as immunological adjuvants and agonistic antibodies. The combination of immune activation with various cancer-ablation techniques enhances the death of tumors.

The photothermal immunotherapy (PTT) technique has also been demonstrated to reduce tumor development and enhance immune response. However, PTT efficiency in clinical applications is hindered by its poor stability, low therapeutic potency, and loss of affinity in the multistep delivery carrier synthesis [60]. A research team led by Zou developed a mesoporous carbon nanocomposite (MCN) loaded with IR792 a near-infrared (NIR) laser dye (IR792-MCN) [61]. Photothermal immunotherapy using NIR laser irradiation is improved by combining the PD-L1 antibody with the nanocarrier (IR792-MCN@ICB). The delivered IR792-MCN@ICB irradiated at 808 nm efficiently kills tumor cells by inducing DC maturation and secreting cytokines. PD-L1 gene silencing in conjunction with photothermal immunotherapy promotes tumor-infiltrating CD8^+^ and CD4^+^ T cells, which reduces tumor growth and prevents cancer metastasis. Cancer immunotherapy based on ICDs in combination with inhibitors as adjuvants plays a significant role in determining the effectiveness of personalized therapy (Figure 6).

## 3. Diagnostic Imaging for Cancer Immunotherapy

**Figure 7:**
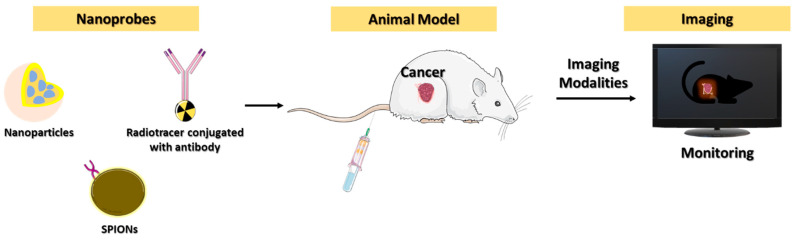
A schematic representation illustrating the nanoparticles’ role in different imaging modalities for cancer immune checkpoint detections.

To improve patient outcomes in the immuno-oncology (IO), existing diagnostic methods, including CT, PET scans, and serum protein biomarkers, are unreliable. In this regard, several point-of-care diagnostic approaches have been developed, but none of these techniques are approved by the FDA to be in clinical practice. IHC is the only standard diagnostic in practice, however, it does not provide complete information for the tumor or metastatic cancers. IC markers from blood can be diagnosed easily, noninvasively, and can be tested multiple times. Imaging technologies play a pivotal role in visualizing cancer regions and revealing treatment responses (Figure 7).

Imaging systems can be classified into structural and molecular modalities. In preclinical and clinical practice, imaging techniques used to detect tumors include computed tomography (CT), ultrasound (US), magnetic resonance imaging (MRI), positron emission tomography (PET), and optical imaging (OI) [62]. PET and OI (visible and NIR-based) show the molecular dynamics of cancer cells. All these imaging modalities have their pros and cons when used for cancer immune checkpoint imaging.

### 3.1. PET Imaging

PET/CT combines the benefits of two imaging techniques to accurately determine the state of the cancer. This hybrid imaging technique provides the complementary structural and molecular information, the localization zone, and 3D volume information with an improved signal-to-noise ratio. Extensive works based on imaging in cancer immunotherapy enable whole-body visualization of the tumor and immune cell characteristics without invasive procedures, which may help in the assessments for ICI therapies. Recently, a large amount of interest was focused on targeting ICIs such as PD-L1, PD-L2, PD-1, and CTLA-4. For instance, conjugating the contrast agents with small molecules, full antibodies, and antibody fragments enables targeted imaging.

PET imaging is often used due to its high sensitivity; however, it is restricted in its spatial resolution. The rise of immune PET imaging is well-regarded for its speed in diagnostic and tumor-progression evaluations [62]. PD-1 is an immune checkpoint that prevents the immune system from attacking healthy cells. However, cancerous cells often express their ligand, PD-L1, as a way of deceiving the body’s natural defense system. PD-1 is expressed in T cells and cancer cells, or antigen-presenting cells will often express PD-L1 as a way of blocking antitumor responses.

**Figure 8:**
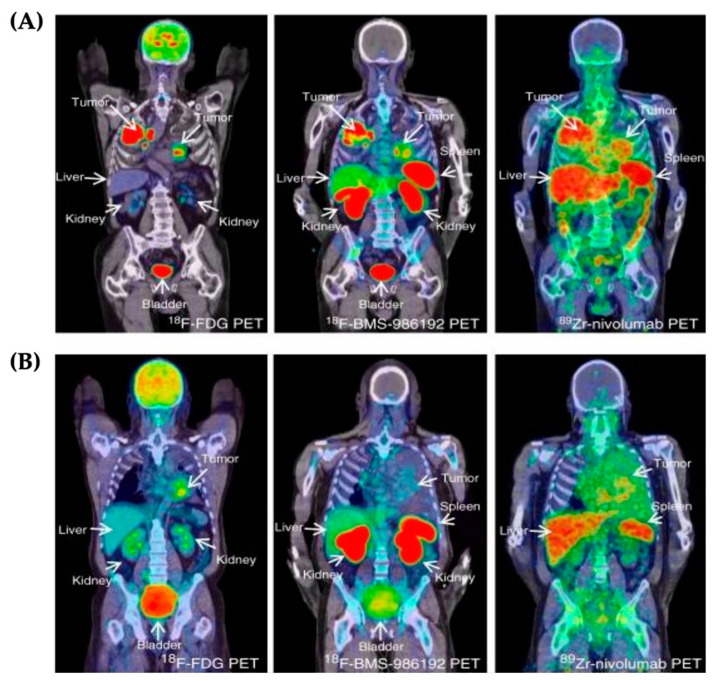
PET/CT images of panel (**A**,**B**) are corresponding from two patients, respectively. These images are ^18^F-FDG PET (left), ^18^F-BMS-986192 PET (center) and ^89^Zr-Nivolumab (right).^18^F-FDG PET scan results show malignancies with significant glucose metabolism in both the lungs and the mediastinal lymph nodes. PET with ^18^F-BMS-986192 reveals uneven tracer uptake within and between tumors. ^18^F-FDG PET scans show that the tumor on the left side has a high glucose metabolism. PET with ^18^F-BMS-986192 indicates little tumor tracer uptake. Nivolumab PET with ^89^Zr indicates diverse tracer uptake in the tumor. Adapted from reference [64], licensed under a CC BY 4.0.

Recently, the ^64^Cu-FN3hPD-L1 binder was engineered for imaging under PET/CT scans. Natarajan and colleagues developed this binder to target the PD-L1 immune checkpoint, using a 12kD human type 3 fibronectin (FN3) scaffold with a copper-64 radiotracer. The binder was tested in vitro using cancerous human xenografts as well as in vivo using mice models. It was found that the ^64^Cu-FN3hPD-L1 binder could travel to the tumor site and provide visualization of the cancer, as well as reveal treatment progress and cancer metastasis [63]. The first immune checkpoint study using in vivo conditions tested two different tracers using PET/CT imaging on the whole body. Before being treated with nivolumab, ^18^fluorine-labeled anti-PD-L1 Adnectin (^18^F-BMS-986192) and ^89^zirconium-labeled nivolumab (^89^Zr-nivolumab) were tested in patients with non-small-cell lung cancer (NSCLC). The ^89^Zr-nivolumab tracer has a large molecular size and therefore was found to be effective when imaged 5–7 days after injection. On the other hand, the ^18^F-BMA-986192 tracer was enhanced when imaged on the same day as injected. Both tracers were found to have significant binding and high-contrast visualization under the PET/CT scans, with no adverse reactions to the tracers (Figure 8). Two of the patients tested had metastasis to the brain, which was not all visible under the scans due to low penetration of the tracers or the low PD-1/PD-L1 expression in the cancer regime [64].

Engineering the antibody with the ^89^Zr tracer facilitates metastasis tracing using an immune-PET/CT scan. The cluster of differentiation 3 (CD3) is an immune checkpoint that acts as a T cell coreceptor and is necessary for T cell activation. CD3 will bind to antigens which are presented on antigen-presenting cells. CD3 will then send a signal back to the nucleus of the T cell for immune activation. Anti-CD3 is an antibody that will bind to CD3 on the surface of T cells and tracking anti-CD3 allows for the visualization of the antigen. Deferoxamine (DFO) was used as a bifunctional chelating agent because of its high chemical and biological stability with ^89^Zr. ^89^Zr-DFO-anti-CD3 was tested in in vitro and in vivo conditions and was found to have a high uptake in the TME. The engineered antibody also demonstrated a rapid clearance time, resulting in clear visualization under the PET/CT scan. The study also found that the administration of ^89^Zr-DFO-anti-CD3 led to a decrease in the CD4^+^ T cell population and an increase in the number of CD8^+^ T cells, although there was no significant change in the total number of T cells. This observation benefits cancer patients, as a high CD8^+^-to-CD4^+^ T cell ratio is associated with better patient prognosis [65].

### 3.2. Near-Infrared Imaging

Near-infrared fluorescence imaging (NIFR) has high sensitivity and specificity, although the penetration depth of the laser holds it back. On the other hand, magnetic resonance imaging (MRI) is known for its high resolution as well as strong contrast in soft tissue images but has also been found to have high false-positive rates. However, it has been discovered that combining the NIFR and MRI techniques can be a strong tool for tumor detection. A study focusing on triple-negative breast cancer (TNBC) used this dual-imaging method to target PD-L1 using a nanoparticle probe. Second-near-infrared (NIR-II) imaging refers to light within the 1000–1700 nm wavelength range, as opposed to near-infrared imaging, which begins around 800 nm [66]. NIR-II provides higher resolution and a deeper penetration depth, allowing for more accurate imaging that provides more functional anatomical information.

Lui and coworkers developed a nanoparticle combining the photosensitizer BODIPY (BDP) and PD-L1 (BDP-I-N-anti-PD-L1) for molecular imaging, photodynamic treatment (PDT), and immunological combination therapy. PDT’s known benefits include the microinvasive eradication of cancers, low adverse effects, and minimum tissue damage. However, this study also emphasized the anticancer effects of PDT on fluorescent nanoparticles. These engineered nanoparticles were tested in vitro and in vivo using mouse models and were demonstrated to be a strong tool for the molecular imaging of MC38 tumors. They also induced the formation of O_2_ to kill the cancerous cells while preventing damage to healthy cells [67].

### 3.3. Magnetic Resonance Imaging

Magnetic resonance imaging (MRI) is a strong imaging technique for nanoparticles using SPIONs. Nanoparticles imaged under MRIs have both magnetic and biodegradable advantageous properties. A bioengineered nanoparticle using SPIONs and siRNA-targeting PD-L1 for theranostics in gastric agents found improved MRI contrast, among other benefits. The MR images showed increased T2-weighted contrast, making it easier to visualize and therefore monitor cancer noninvasively [68]. Superparamagnetic iron oxide nanoparticles (SPIONs) possess improved magnetization properties, as well as biocompatibility and stability in aqueous solutions. SPIONS were used as a contrast agent for MRI and conjugated with Cy5.5 dye for the NIRF. The SPIONs-L1-Cy5.5 probe was tested in vitro and in vivo conditions. Since PD-L1 is upregulated in TNBC and associated with poor patient prognosis, its imaging can prevent oncologists from stopping immunotherapy when it could be helpful. The synthesized dual-mode imaging probe was able to detect PD-L1 cancer and exhibited slight cytotoxicity [69].

### 3.4. Challenges and Perspectives of Cancer Immunotherapy Imaging

Medical imaging modalities are useful for imaging immune checkpoints. Multimodal approaches are typically the most successful. For example, PET/CT scans allow for the visualization of metabolic activity with the added benefit of seeing the structural anatomy using CT. These imaging techniques visualize the cancer’s solid condition and metastatic regime to identify affected organs. Combining NIFR imaging with MRIs improves molecular sensitivity and specificity. The MRI offers more in-depth complementary structural details during in vivo conditions. Combining imaging modalities with nanoparticles as a contrast agent improves cancer immunotherapy detection and patient prognosis. Ultrasounds are gaining recognition in the field because of their low cost with no radiation hazards. Microbubbles as contrast agents can target immune checkpoints and are captured using ultrasound imaging, which has better economic benefits over other techniques. Currently, the cancer immunotherapy treatment success rate is ~20% [70], while the remaining patients were treated with traditional and painful invasive treatments, which carries negative side effects and discomfort to patients. Therefore, there is an urgent need to study these immune-modulated drugs before their clinical applications. In this regard, additional diagnostic tools are required to monitor and measure the level of cancer markers for drug interaction and treatment outcomes. For example, peptides, protein binders, and antibodies-based tracers were developed and tested for immune checkpoint imaging to measure the expression level in the TME. Systemically small protein binders are cleared much faster than high-molecular-weight antibodies.

## 4. Lab-on-Chip-Based Theragnosis for Cancer Immunotherapy

Polydimethylsiloxane (PDMS) lab-on-chips (LOCs) are widely utilized in laboratories for fabricating microchannels and microfluidic devices due to their low cost and ease of production. Glass is another commonly utilized material due to its transparency, suitability with micrometer-scale machining, and chemical inertness, which permits diverse electrode integration. Silicon on the other hand is rarely utilized over glass polymers because of its high electroconductivity, which is not ideal for high-voltage tests, and its high expense [71]. Paper-based LOCs (P-LOCs) are mostly used for the detection of chemicals and compounds, along with a few other applications. P-LOCs provide the most ecofriendly and inexpensive device, which may have strong outcomes for applications requiring ultra-low costs. Toxin and pathogen detection, as well as glucose, immunological molecule, and protein concentration, are just some of the uses of P-LOCs [72]. When taking the cost, the applicability in point-of-care diagnostics, and the reproducibility, PDMS is the desired material for most labs to test nanomaterials for immunotherapy. PDMS improves visualization of nanoparticles, quantum dots, and nanowires while being tested on the TME, offers better biocompatibility, allowing enhanced sensitivity and biolabeling, and allows for proper gas permeability, which all contribute to the ability of LOCs to improve cell culture and TME analysis.

Imaging, quantification, and recurrent testing are critical in the field of immunotherapy to establish a treatment’s efficacy. Successfully validating a drug’s effectiveness takes into account the interactions between the drug and disease and measures the specificity in a realistic model. During the past few decades, the space of immunotherapy drug development, testing, and translation have made tremendous strides. However, with each drug costing an average of USD 1–2 billion and taking 10–15 years to be approved for clinical applications, most of which are not as widely effective in the patient setting, the ability for patients to receive a potentially life-saving treatment is not optimistic. There is a lack of confidence in translating preclinical findings of immune-based therapies for any given patient.

For testing, traditional animal and in vitro methods of cancer modeling cannot recapitulate the human TME. Currently, some factors that contribute to the challenge of drug development include (i) animal models often cannot recapitulate an entire disorder or disease; (ii) the heterogeneity of the patient population cannot be considered. Even within a single patient, the TME differs greatly.

Studying the TME using microfluidics platforms for both solid and hematological cancers is possible through LOC microfluidic devices [73]. LOCs can be characterized as an organization of electrodes, microchannels, and sensors. It presents the possibility to study different cell types without the use of fluorescent or magnetic labeling. Instead, it incorporates microfluidic technology such as pressure, capillary flow, or electrokinetic effects with detectors for optical analysis. This enables LOCs to recapitulate the complex and dynamic cancer–immune system interaction, providing a new way to conduct a host-like preclinical evaluation of immunotherapeutic strategies, such as immune checkpoint blockade therapy, to allow a more patient-specific understanding of a tumor’s biology [74].

The LOC’s small size (cm) and user-friendly interface provide a way of integrating thousands of biochemical operations using as little as a drop of blood [75]. This can be attributed to the spatial constraint in LOCs, as it translates to considerably reduced convective mixing in no-flow systems, hence losing less sample volume. Most of the movement of molecules is instead powered by diffusion. The practicality of the LOC, because of its small size, includes its ability to analyze data comparable to analyses conducted in a fully equipped analytical laboratory. As of recently, the LOC places a focused look at human data that might lead to improved target identification and validation.

The standard mechanism of LOCs includes the sample being passed through a network of micrometer-sized channels fabricated on a surface compatible with the sample. With these channels, handling and preparing the sample, along with the analysis of that sample, can all be done without having to change the chip. In addition, the rate of flow of the sample of interest passed through the device is closely controlled. The LOC allows a more cost-effective and user-friendly alternative for single-cell analysis (SCA). For example, with single-cell analysis, conventional flow cytometry, which requires the use of high volumes of samples and reagents, is inferior to the LOC microfluidics device.

### 4.1. Multilayered Blood Vessel/Tumor Tissue Chip

Immunotherapy recipients are carefully chosen based on a number of criteria, including microsatellite instability (MSI) status, PD-L1 expression, and the number of somatic mutations present in the patient’s tumor. Because these biomarkers are associated with positive immunotherapy results, investigating the TME for indicators such as levels of PD-L1 expression has become the cornerstone of predicting immunotherapy success for a particular patient. Selection for these patients leaves only a fraction of eligible candidates. However, a faster, less invasive, and repeatable method of assessment would increase the effectiveness and even expand the patient population who get to receive immunotherapy. Additionally, T cells must extravasate from the blood vessels to reach the site of the tumor and then interstitially migrate into tumors. Being able to observe the mechanism in real-time and understand how a therapy interacts with patient-specific cellular environments would expand and expedite the patient selection process, thereby providing more people with life-saving treatments.

The multilayered blood vessel/tumor tissue chip (MBTC) demonstrated the full series of T cell/tumor infiltration, including extravasation and interstitial migration. The MBTC is derived from a parallel plate flow chamber, which is an in vitro model that recapitulates the fluid shear stress on cells that are naturally exposed to dynamic fluid flow. A porous membrane covered with an endothelial cell monolayer mimics a host-like environment and is referred to as the top fluidic chamber. Additionally, a collagen gel block encapsulates the tumor cell and is referred to as the matrix. Systematic investigation of T cell/tumor infiltration and T cell dynamics in TMEs are recapitulated in the MBTC. Preclinical evaluation of immunotherapies was demonstrated by testing anti-vascular endothelial growth factor (anti-VEGF) treatment on an MBTC. VEGF is produced by T cells and suppresses the endothelial cell activation by cytokines or by inducing endothelial cell anergy, which refuses T cell extravasation. Anti-VEGF is a treatment used to reverse the effects of VEGF, thereby promoting T cell infiltration. Using the MBTC, Lee demonstrated that the endothelial cell energy could be reversed by blocking VEGF secretion. The MBTC was concluded to show how tumor cells can increase extravasation by induced endothelial cell anergy and can promote the interstitial migration of T cells into tumors by producing chemokines to recruit T cells [76].

### 4.2. Chimeric Antigen Receptor (CAR)-T LOC

Deficient TCR affinity and variable target toxicity are some of the limitations of immunotherapy, as low levels of expression of tumor antigens coupled with poor specific TCRs make it so that target sites are less likely to be bound. Chimeric antigen receptor cell therapy (CAR)-T is a way of genetically altering a host’s T cells in vitro to target many hematological malignancies. However, it is not as efficacious when targeting solid tumors. With CAR-T cell therapy having an overwhelming cost average of USD 1,000,000, and the 2D in vitro models failing to recapitulate the TME, a miniaturized method of CAR-T cell therapy assessment using 3D solid tumor models would be a more effective strategy [77].

The multichannel microfluidic immunoassay was developed on a single chip and used for the evaluation of CAR-T cell cytotoxicity and targeting specificity on 3D cancer spheroids [78]. Using the lab-on-chip, the monitoring of the interaction between CAR-T cells and spheroid cocultures demonstrated that CAR-T cells migrate to target-expressing cancer cells to elicit a cytotoxic effect. A combination of CAR-T cells with other drug therapies determined that CAR-T cell cytotoxicity is enhanced with therapeutic drugs of anti-PD-L1 and carboplatin. LOC-based experiments propose a material-saving and better preclinical screening tool for assessing immunotherapy.

### 4.3. D/3D Cell Culture Model for Better Cancer Immunotherapy

**Figure 9:**
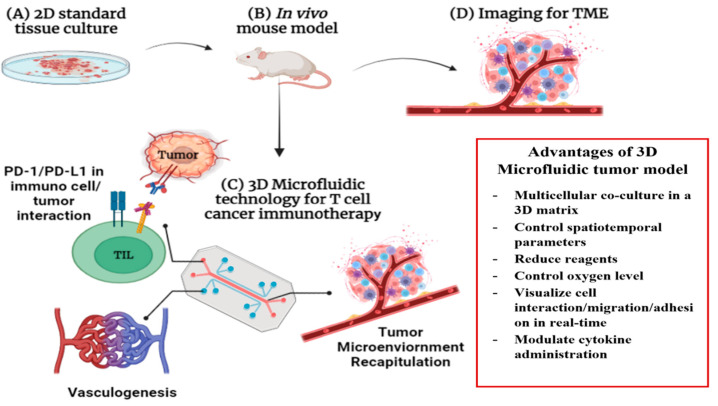
The 3D cell culture model for immunotherapy compared to standard 2D cell tissue culture. (**A**) The typical 2D culture grown as a monolayer in a flat petri dish, attached to a plastic surface. (**B**) In vivo mouse model to test and validate immunotherapeutic response in real TME. (**C**) Full recapitulation of the TME using human tissue layers to line 3D lab on chip microfluidic device with proper vasculogenesis. PD-1/PD-L1 interaction with tumor-infiltrating lymphocyte. (**D**) Standard imaging for analysis of TME interactions with immunotherapeutics for in vivo models, 2D cultures and 3D microfluidics. Modified from Ref. [78].

The standard 2D culture has been the gold standard in cancer biology for many years; however, it is not representative of the real TME because cells are grown in a petri dish. Two-dimensional systems are unable to integrate the structural and mechanical properties that make up the TME, which provides an inaccurate understanding of a drug’s function. However, 3D microfluidic culture systems are being incorporated into the laboratory. In particular, in vitro microfluidic 3D models are more precise and realistic representations of the host environment, creating a more human-like environment for the cells to grow in (Figure 9).

**Figure 10:**
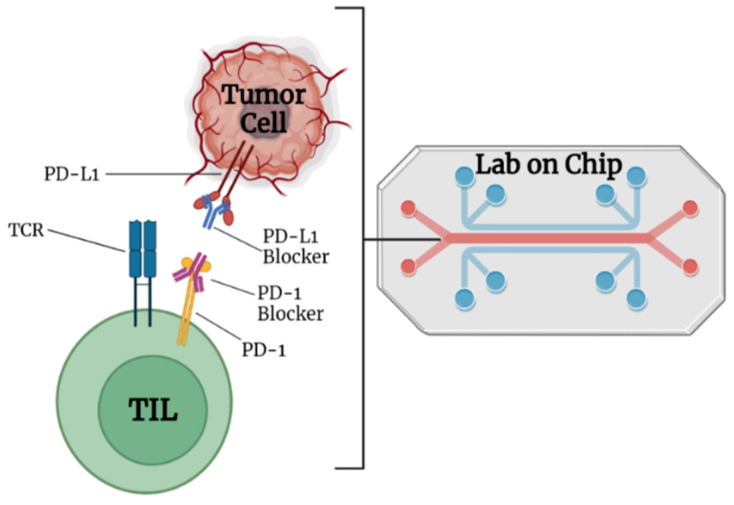
Programmed death-1 (PD-1) and programmed death-1 ligand (PD-L1)-blocker antibodies binding to their respective sites on tumor-infiltrating lymphocytes and tumor cells are shown in a lab-on-chip microfluidic device.

A novel method of profiling the response to the PD-1 blockade therapy was studied using organotypic tumor spheroids cultured in collagen hydrogels placed in a commercially available 3D microfluidic device, the DAX-1 3D cell culture chip [79]. In addition to PD-1 inhibition, there is a need to assess the sensitivity of murine/patient-derived organotypic tumor spheroids (MDOTS/PDOTS) to combination treatments. MDOTS/PDOTS was added into a neutral pH collagen solution with a volume that was adjusted to retain 10–20 thousand cells per microfluidic chamber. Using the murine tumor model, the authors show the sensitivity to ICB ex vivo with light/phase contrast microscopy, time-lapse (live) imaging, immunofluorescence microscopy, live/dead imaging, and secreted cytokine profiling. A disadvantage shared by 3D culture systems is the need to separate single cells from spheroid structures by the proteolytic degradation of single layers, prolonging the time needed to run the analysis. Addressing this problem in future LOCs would increase its scope in point-of-care testing (Figure 10).

### 4.4. Challenges and Perspectives of LOC

Currently, the space of the LOC has revolutionized the realm of point-of-care testing and carrying this expansion in LOC technology is promising. However, there are challenges with the mass production of chips of the same quality. Cost for production is a limiting factor (still overall less expensive) because most LOCs are of one time-usage. To combat these challenges, recycled materials such as CDs to make at-home LOCs with the same accuracy as commercially bought LOCs are being developed. With many more developments to come, the multianalyte detection capabilities of LOCs are evidence of a gateway to more accessible, precise, and cost-effective methods for immunotherapy testing.

The current methods of imaging, analyzing, and encapsulating the TME with antibody-based immunotherapy ramp-up costs and diminish reproducibility. Animal-based models are utilized for observing the effects of PD-1/PD-L1, CTLA-4, or VEGF antibody treatments on solid tumors and hematological cancers, which, when paired with radiolabeling for PET/CT or MRI, take longer and can cloud experiments with many confounding variables. The LOC greatly expedites the time-to-image and analysis results for antibody treatment and other nanoparticle-type treatments. One challenge with LOCs is the ability to analyze the large compilation of bioimages taken from the recapitulated TME. Recently, a strategy for analyzing the infection and killing of cancer cells using the oncolytic vaccinia virus (OVV) was proposed. Using advanced algorithms along with appropriate analysis, the synergistic cooperation of the OVV and immune cells to kill cancer cells was uncovered.

## 5. AI and ML in Immune-Targeted Drug Delivery

Artificial intelligence is a software tool to obtain sensitive information better than can be achieved by human intelligence. In the medical community, AI can be effectively used for generating disease and treatment models with the existing data, which can produce high-throughput information to predict cancer cures. For instance, AI tools in medicine provide suggestions on critical decision-making and interpreting the given data, thereby generating visual representations to enhance clinicians’ knowledge [80]. The advancement of cancer treatment and the prediction of personalized medicine was the primary focus of AI researchers [81]. Radionics is an AI tool developed for the oncology-related analysis of medical images to recognize tumor regions precisely. This technology helps physicians to know how to perform a biopsy to obtain overall tumor regions before surgery. Currently, AI is used in medical imaging modalities to delineate and segment tumors from normal regions via imaging agents. Moreover, machine learning and deep learning algorithms and methodologies are applied in medical images for automated labeling and annotation to assist the routine workflow of physicians. In the future, medical imaging technologies enabled with AI/ML tools will be a revolutionizing strategy to monitor ICI-based therapy and treatment response. Though methodologies and techniques used for cancer immunotherapy are efficient, the variations in the treatment efficacy are still undetermined. Post-cancer immunotherapy treatment affects the patient’s incidence of immune-related problems such as autoimmune disorders that lead to organ dysfunction. In the future, AI can be helpful for the clinician to predict personalized treatment options for effective immunotherapy to cure cancer without any side effects.

## 6. Conclusions and Future Directions

The most important advantage of immunotherapy is the that immune system can remember the disease, allowing it to detect and destroy tumor variants as they emerge. This attribute will always be an inherent advantage over other cancer therapies. Currently, the focus of cancer immunotherapy treatment has moved to treat the specific tumor characteristics and their interaction with the intrinsic immune system instead of treating the diseased organ. The good news is that several immunotherapy drugs are now rapidly being approved to treat multiple cancers. This may be used either as first-line treatment or when standard first-line treatment has failed. For instance, the usage of the FDA-approved anti-PD-1, and pembrolizumab drugs for the treatment of unresectable tumor conditions like high MSI or mismatch-repair–deficient has increased patients’ therapeutic options. Patients with solid tumor recurrence who have exhausted all other therapy options are satisfied with this treatment choice. In addition, this is the first time the FDA has approved a cancer treatment based on a common biomarker rather than the location of the tumor.

However, active research is needed to understand why some cancers and patients respond so well to immunotherapy while others do not. This may be due to tumors becoming resistant after the initial response. A cancer immunotherapy approach must find ways to manipulate the immune system in patients who do not exhibit an immune response to their tumors. TILs need to infiltrate into the TME for immunotherapy to be effective [82]. Furthermore, by understanding the immunology of the host and tumor, we will hopefully be able to identify more specific diagnostic biomarkers.

In the future, personalized medicine, with novel combinations of therapy and new treatment sequences, could provide better cures for cancer. In addition, a better understanding of resistance and recurrence will help effectively control the disease and promote the long-term survival of the patient. For example, a combination of ICB and CAR-T cell therapy is surging for noninvasive effective cancer treatment [83]. Overall, these novel approaches, including immuno nano platforms, may provide better treatment options and opportunities to prolong survival and improve the quality of the patient’s well-being from cancer.

## Data Availability

Data available on request due to restrictions, e.g., privacy or ethical.

## References

[1] Burkholder B., Huang R.Y., Burgess R., Luo S., Jones V.S., Zhang W., Lv Z.Q., Gao C.Y., Wang B.L., Zhang Y.M. (2014). Tumor-induced perturbations of cytokines and immune cell networks. Biochim. Biophys. Acta.

[2] Gonzalez H., Hagerling C., Werb Z. (2018). Roles of the immune system in cancer: From tumor initiation to metastatic progression. Genes Dev..

[3] Kartikasari A.E.R., Huertas C.S., Mitchell A., Plebanski M. (2021). Tumor-Induced Inflammatory Cytokines and the Emerging Diagnostic Devices for Cancer Detection and Prognosis. Front. Oncol..

[4] Vesely M.D., Schreiber R.D. (2013). Cancer immunoediting: Antigens, mechanisms, and implications to cancer immunotherapy. Ann. N. Y. Acad. Sci..

[5] Pucci C., Martinelli C., Ciofani G. (2019). Innovative approaches for cancer treatment: Current perspectives and new challenges. Ecancermedicalscience.

[6] Zahavi D., Weiner L. (2020). Monoclonal Antibodies in Cancer Therapy. Antibodies.

[7] Nixon N.A., Blais N., Ernst S., Kollmannsberger C., Bebb G., Butler M., Smylie M., Verma S. (2018). Current landscape of immunotherapy in the treatment of solid tumors, with future opportunities and challenges. Curr. Oncol..

[8] Kirilovsky A., Marliot F., El Sissy C., Haicheur N., Galon J., Pages F. (2016). Rational bases for the use of the Immunoscore in routine clinical settings as a prognostic and predictive biomarker in cancer patients. Int. Immunol..

[9] Li K., Luo H., Huang L., Luo H., Zhu X. (2020). Microsatellite instability: A review of what the oncologist should know. Cancer Cell Int..

[10] Fan S., Ren H., Zhao L., Yin J., Feng G., Wang J., Guan H. (2020). Neurological immune-related adverse events associated with immune checkpoint inhibitors: A review of the literature. Asia Pac. J. Clin. Oncol..

[11] Nishino M., Ramaiya N.H., Hatabu H., Hodi F.S. (2017). Monitoring immune-checkpoint blockade: Response evaluation and biomarker development. Nat. Rev. Clin. Oncol..

[12] Patrinely J.R., Johnson R., Lawless A.R., Bhave P., Sawyers A., Dimitrova M., Yeoh H.L., Palmeri M., Ye F., Fan R. (2021). Chronic Immune-Related Adverse Events Following Adjuvant Anti-PD-1 Therapy for High-risk Resected Melanoma. JAMA Oncol..

[13] Galluzzi L., Chan T.A., Kroemer G., Wolchok J.D., Lopez-Soto A. (2018). The hallmarks of successful anticancer immunotherapy. Sci. Transl. Med..

[14] Bai R., Chen N., Li L., Du N., Bai L., Lv Z., Tian H., Cui J. (2020). Mechanisms of Cancer Resistance to Immunotherapy. Front. Oncol..

[15] Rameshbabu S., Labadie B.W., Argulian A., Patnaik A. (2021). Targeting Innate Immunity in Cancer Therapy. Vaccines.

[16] Gavas S., Quazi S., Karpinski T.M. (2021). Nanoparticles for Cancer Therapy: Current Progress and Challenges. Nanoscale Res. Lett..

[17] Anselmi M., Borbely A., Figueras E., Michalek C., Kemker I., Gentilucci L., Sewald N. (2021). Linker Hydrophilicity Modulates the Anticancer Activity of RGD-Cryptophycin Conjugates. Chemistry.

[18] Li Q., Li X., Zhao C. (2020). Strategies to Obtain Encapsulation and Controlled Release of Small Hydrophilic Molecules. Front. Bioeng. Biotechnol..

[19] Subhan M.A., Yalamarty S.S.K., Filipczak N., Parveen F., Torchilin V.P. (2021). Recent Advances in Tumor Targeting via EPR Effect for Cancer Treatment. J. Pers. Med..

[20] Tan S., Li D., Zhu X. (2020). Cancer immunotherapy: Pros, cons and beyond. Biomed. Pharmacother..

[21] Teixidor E., Bosch-Barrera J. (2019). the dark side of immunotherapy: Challenges facing the new hope in cancer treatment. Ann. Transl. Med..

[22] Chevolet I., Speeckaert R., Schreuer M., Neyns B., Krysko O., Bachert C., Hennart B., Allorge D., van Geel N., van Gele M. (2015). Characterization of the in vivo immune network of IDO, tryptophan metabolism, PD-L1, and CTLA-4 in circulating immune cells in melanoma. Oncoimmunology.

[23] Peer D., Karp J.M., Hong S., Farokhzad O.C., Margalit R., Langer R. (2007). Nanocarriers as an emerging platform for cancer therapy. Nat. Nanotechnol..

[24] He X., Xu C. (2020). Immune checkpoint signaling and cancer immunotherapy. Cell Res..

[25] Mokhtari R.B., Sambi M., Qorri B., Baluch N., Ashayeri N., Kumar S., Cheng H.M., Yeger H., Das B., Szewczuk M.R. (2021). The Next-Generation of Combination Cancer Immunotherapy: Epigenetic Immunomodulators Transmogrify Immune Training to Enhance Immunotherapy. Cancers.

[26] Qin S., Xu L., Yi M., Yu S., Wu K., Luo S. (2019). Novel immune checkpoint targets: Moving beyond PD-1 and CTLA-4. Mol. Cancer.

[27] Greenbaum U., Mahadeo K.M., Kebriaei P., Shpall E.J., Saini N.Y. (2020). Chimeric Antigen Receptor T-Cells in B-Acute Lymphoblastic Leukemia: State of the Art and Future Directions. Front. Oncol..

[28] Yetisgin A.A., Cetinel S., Zuvin M., Kosar A., Kutlu O. (2020). Therapeutic Nanoparticles and Their Targeted Delivery Applications. Molecules.

[29] Yu X., Fang C., Zhang K., Su C. (2022). Recent Advances in Nanoparticles-Based Platforms Targeting the PD-1/PD-L1 Pathway for Cancer Treatment. Pharmaceutics.

[30] Reda M., Ngamcherdtrakul W., Nelson M.A., Siriwon N., Wang R., Zaidan H.Y., Bejan D.S., Reda S., Hoang N.H., Crumrine N.A. (2022). Development of nanoparticle-based immunotherapy targeting PD-L1 and PLK1 for lung cancer treatment. Nat. Commun..

[31] Xiao Y., Chen J., Zhou H., Zeng X., Ruan Z., Pu Z., Jiang X., Matsui A., Zhu L., Amoozgar Z. (2022). Combining p53 mRNA nanotherapy with immune checkpoint blockade reprograms the immune microenvironment for effective cancer therapy. Nat. Commun..

[32] Mills C.N., Nowsheen S., Bonner J.A., Yang E.S. (2011). Emerging roles of glycogen synthase kinase 3 in the treatment of brain tumors. Front. Mol. Neurosci..

[33] Kulikov R., Boehme K.A., Blattner C. (2005). Glycogen synthase kinase 3-dependent phosphorylation of Mdm2 regulates p53 abundance. Mol. Cell Biol..

[34] Badiee P., Maritz M.F., Thierry B. (2022). Glycogen kinase 3 inhibitor nanoformulation as an alternative strategy to inhibit PD-1 immune checkpoint. Int. J. Pharm..

[35] Waldman A.D., Fritz J.M., Lenardo M.J. (2020). A guide to cancer immunotherapy: From T cell basic science to clinical practice. Nat. Rev. Immunol..

[36] Yang L., Shi P., Zhao G., Xu J., Peng W., Zhang J., Zhang G., Wang X., Dong Z., Chen F. (2020). Targeting cancer stem cell pathways for cancer therapy. Signal Transduct. Target Ther..

[37] Dasari S., Tchounwou P.B. (2014). Cisplatin in cancer therapy: Molecular mechanisms of action. Eur. J. Pharmacol..

[38] Yao H., Shen N., Ji G., Huang J., Sun J., Wang G., Tang Z., Chen X. (2022). Cisplatin Nanoparticles Promote Intratumoral CD8(+) T Cell Priming via Antigen Presentation and T Cell Receptor Crosstalk. Nano Lett..

[39] Bansal-Pakala P., Halteman B.S., Cheng M.H., Croft M. (2004). Costimulation of CD8 T cell responses by OX40. J. Immunol..

[40] Liu Z., Han C., Fu Y.X. (2020). Targeting innate sensing in the tumor microenvironment to improve immunotherapy. Cell Mol. Immunol..

[41] Dane E.L., Belessiotis-Richards A., Backlund C., Wang J., Hidaka K., Milling L.E., Bhagchandani S., Melo M.B., Wu S., Li N. (2022). STING agonist delivery by tumor-penetrating PEG-lipid nanodiscs primes robust anticancer immunity. Nat. Mater..

[42] Nakamura T., Yamada K., Sato Y., Harashima H. (2020). Lipid nanoparticles fuse with cell membranes of immune cells at low temperatures leading to the loss of transfection activity. Int. J. Pharm..

[43] Khalifa A.M., Nakamura T., Sato Y., Sato T., Hyodo M., Hayakawa Y., Harashima H. (2022). Interval- and cycle-dependent combined effect of STING agonist loaded lipid nanoparticles and a PD-1 antibody. Int. J. Pharm..

[44] Messenheimer D.J., Jensen S.M., Afentoulis M.E., Wegmann K.W., Feng Z., Friedman D.J., Gough M.J., Urba W.J., Fox B.A. (2017). Timing of PD-1 Blockade Is Critical to Effective Combination Immunotherapy with Anti-OX40. Clin. Cancer Res..

[45] Guo W., Wu Z., Chen J., Guo S., You W., Wang S., Ma J., Wang H., Wang X., Wang H. (2022). Nanoparticle delivery of miR-21-3p sensitizes melanoma to anti-PD-1 immunotherapy by promoting ferroptosis. J. Immunother. Cancer.

[46] Liu J., Fu M., Wang M., Wan D., Wei Y., Wei X. (2022). Cancer vaccines as promising immuno-therapeutics: Platforms and current progress. J. Hematol. Oncol..

[47] Pallerla S., Abdul A., Comeau J., Jois S. (2021). Cancer Vaccines, Treatment of the Future: With Emphasis on HER2-Positive Breast Cancer. Int. J. Mol. Sci..

[48] Chen B.Q., Zhao Y., Zhang Y., Pan Y.J., Xia H.Y., Kankala R.K., Wang S.B., Liu G., Chen A.Z. (2023). Immune-regulating camouflaged nanoplatforms: A promising strategy to improve cancer nano-immunotherapy. Bioact. Mater..

[49] Hassannia H., Chaleshtari M.G., Atyabi F., Nosouhian M., Masjedi A., Hojjat-Farsangi M., Namdar A., Azizi G., Mohammadi H., Ghalamfarsa G. (2020). Blockage of immune checkpoint molecules increases T-cell priming potential of dendritic cell vaccine. Immunology.

[50] Barshidi A., Karpisheh V., Noukabadi F.K., Kiani F.K., Mohammadi M., Afsharimanesh N., Ebrahimi F., Kiaie S.H., Navashenaq J.G., Hojjat-Farsangi M. (2022). Dual Blockade of PD-1 and LAG3 Immune Checkpoints Increases Dendritic Cell Vaccine Mediated T Cell Responses in Breast Cancer Model. Pharm. Res..

[51] Jorgovanovic D., Song M., Wang L., Zhang Y. (2020). Roles of IFN-gamma in tumor progression and regression: A review. Biomark Res..

[52] Shofolawe-Bakare O.T., Stokes L.D., Hossain M., Smith A.E., Werfel T.A. (2020). Immunostimulatory biomaterials to boost tumor immunogenicity. Biomater. Sci..

[53] Yao X., Ma S., Peng S., Zhou G., Xie R., Jiang Q., Guo S., He Q., Yang W. (2020). Zwitterionic Polymer Coating of Sulfur Dioxide-Releasing Nanosystem Augments Tumor Accumulation and Treatment Efficacy. Adv. Healthc. Mater..

[54] Weiss A.M., Hossainy S., Rowan S.J., Hubbell J.A., Esser-Kahn A.P. (2022). Immunostimulatory Polymers as Adjuvants, Immunotherapies, and Delivery Systems. Macromolecules.

[55] Formenti S.C., Demaria S. (2009). Systemic effects of local radiotherapy. Lancet Oncol..

[56] Wang Y., Deng W., Li N., Neri S., Sharma A., Jiang W., Lin S.H. (2018). Combining Immunotherapy and Radiotherapy for Cancer Treatment: Current Challenges and Future Directions. Front. Pharmacol..

[57] Hagan C.T.t., Mi Y., Knape N.M., Wang A.Z. (2020). Enhancing Combined Immunotherapy and Radiotherapy through Nanomedicine. Bioconjug. Chem..

[58] Lerner E.C., Edwards R.M., Wilkinson D.S., Fecci P.E. (2022). Laser ablation: Heating up the anti-tumor response in the intracranial compartment. Adv. Drug Deliv. Rev..

[59] Deng B., Ma B., Ma Y., Cao P., Leng X., Huang P., Zhao Y., Ji T., Lu X., Liu L. (2022). Doxorubicin and CpG loaded liposomal spherical nucleic acid for enhanced Cancer treatment. J. Nanobiotechnol..

[60] Zhou B., Wu Q., Wang M., Hoover A., Wang X., Zhou F., Towner R.A., Smith N., Saunders D., Song J. (2020). Immunologically modified MnFe2O4 nanoparticles to synergize photothermal therapy and immunotherapy for cancer treatment. Chem. Eng. J..

[61] Wang Y., Wang H., Song Y., Lv M., Mao Y., Song H., Wang Y., Nie G., Liu X., Cui J. (2022). IR792-MCN@ZIF-8-PD-L1 siRNA drug delivery system enhances photothermal immunotherapy for triple-negative breast cancer under near-infrared laser irradiation. J. Nanobiotechnol..

[62] Pan X., Gao A., Lin Z. (2022). Fluorescence imaging of tumor immune contexture in immune checkpoint blockade therapy. Int. Immunopharmacol..

[63] Natarajan A., Patel C.B., Ramakrishnan S., Panesar P.S., Long S.R., Gambhir S.S. (2019). A Novel Engineered Small Protein for Positron Emission Tomography Imaging of Human Programmed Death Ligand-1: Validation in Mouse Models and Human Cancer Tissues. Clin. Cancer Res..

[64] Niemeijer A.N., Leung D., Huisman M.C., Bahce I., Hoekstra O.S., van Dongen G., Boellaard R., Du S., Hayes W., Smith R. (2018). Whole body PD-1 and PD-L1 positron emission tomography in patients with non-small-cell lung cancer. Nat. Commun..

[65] Vera D.R.B., Smith C.C., Bixby L.M., Glatt D.M., Dunn S.S., Saito R., Kim W.Y., Serody J.S., Vincent B.G., Parrott M.C. (2018). Immuno-PET imaging of tumor-infiltrating lymphocytes using the zirconium-89 radiolabeled anti-CD3 antibody in immune-competent mice bearing syngeneic tumors. PLoS ONE.

[66] Xie N., Hou Y., Wang S., Ai X., Bai J., Lai X., Zhang Y., Meng X., Wang X. (2022). Second near-infrared (NIR-II) imaging: A novel diagnostic technique for brain diseases. Rev. Neurosci..

[67] Liu Q., Tian J., Tian Y., Sun Q., Sun D., Wang F., Xu H., Ying G., Wang J., Yetisen A.K. (2021). Near-Infrared-II Nanoparticles for Cancer Imaging of Immune Checkpoint Programmed Death-Ligand 1 and Photodynamic/Immune Therapy. ACS Nano.

[68] Luo X., Peng X., Hou J., Wu S., Shen J., Wang L. (2017). Folic acid-functionalized polyethyleneimine superparamagnetic iron oxide nanoparticles as theranostic agents for magnetic resonance imaging and PD-L1 siRNA delivery for gastric cancer. Int. J. Nanomed..

[69] Li X., Ji Y., Chen M., Zhang S., Wang Z., Su D., Luo N. (2022). A Dual-Mode Imaging Nanoparticle Probe Targeting PD-L1 for Triple-Negative Breast Cancer. Contrast Media Mol. Imaging.

[70] Mellman I., Coukos G., Dranoff G. (2011). Cancer immunotherapy comes of age. Nature.

[71] Nguyen N.T., Shaegh S.A., Kashaninejad N., Phan D.T. (2013). Design, fabrication, and characterization of drug delivery systems based on lab-on-a-chip technology. Adv. Drug Deliv. Rev..

[72] Qamar A.Z., Shamsi M.H. (2020). Desktop Fabrication of Lab-On-Chip Devices on Flexible Substrates: A Brief Review. Micromachines.

[73] Parlato S., Grisanti G., Sinibaldi G., Peruzzi G., Casciola C.M., Gabriele L. (2021). Tumor-on-a-chip platforms to study cancer-immune system crosstalk in the era of immunotherapy. Lab Chip.

[74] Zheng F., Xiao Y., Liu H., Fan Y., Dao M. (2021). Patient-Specific Organoid and Organ-on-a-Chip: 3D Cell-Culture Meets 3D Printing and Numerical Simulation. Adv. Biol..

[75] Arshavsky-Graham S., Segal E. (2022). Lab-on-a-Chip Devices for Point-of-Care Medical Diagnostics. Adv. Biochem. Eng. Biotechnol..

[76] Lee J., Kim S.E., Moon D., Doh J. (2021). A multilayered blood vessel/tumor tissue chip to investigate T cell infiltration into solid tumor tissues. Lab Chip.

[77] Paterson K., Paterson S., Mulholland T., Coffelt S.B., Zagnoni M. (2022). Assessment of CAR-T Cell-Mediated Cytotoxicity in 3D Microfluidic Cancer Co-Culture Models for Combination Therapy. IEEE Open J. Eng. Med. Biol..

[78] Adriani G., Pavesi A., Tan A.T., Bertoletti A., Thiery J.P., Kamm R.D. (2016). Microfluidic models for adoptive cell-mediated cancer immunotherapies. Drug Discov. Today.

[79] Aref A.R., Campisi M., Ivanova E., Portell A., Larios D., Piel B.P., Mathur N., Zhou C., Coakley R.V., Bartels A. (2018). 3D microfluidic ex vivo culture of organotypic tumor spheroids to model immune checkpoint blockade. Lab Chip.

[80] Xu Z., Wang X., Zeng S., Ren X., Yan Y., Gong Z. (2021). Applying artificial intelligence for cancer immunotherapy. Acta Pharm. Sin. B.

[81] Shimizu H., Nakayama K.I. (2020). Artificial intelligence in oncology. Cancer Sci..

[82] Gajewski T.F. (2015). The Next Hurdle in Cancer Immunotherapy: Overcoming the Non-T-Cell-Inflamed Tumor Microenvironment. Semin. Oncol..

[83] Hegde P.S., Karanikas V., Evers S. (2016). The Where, the When, and the How of Immune Monitoring for Cancer Immunotherapies in the Era of Checkpoint Inhibition. Clin. Cancer Res..

